# Experimental and theoretical evaluations on Oleuropein as a natural origin corrosion inhibitor for copper in acidic environment

**DOI:** 10.1038/s41598-022-11598-7

**Published:** 2022-05-09

**Authors:** M. A. Deyab, Q. Mohsen, E. Bloise, M. R. Lazzoi, G. Mele

**Affiliations:** 1 Egyptian Petroleum Research Institute (EPRI), Nasr City, Cairo, Egypt; 2 Department of Chemistry, College of Sciences, Taif University, Taif, Saudi Arabia; 3 Department of Engineering for Innovation, University of Salento, via Monteroni km 1, 73100 Lecce, Italy

**Keywords:** Materials science, Electrochemistry

## Abstract

Copper corrosion in acidic cleaning solutions is a major worry for heat exchangers. Corrosion inhibitors derived from natural sources might be a viable option. The isolation of Oleuropein compound from olive leaf and investigation of its anticorrosion potential for copper in 1.0 M H_2_SO_4_ solution are reported here. All experimental results from LC–MS, FT-IR, ^1^H and ^13^C-NMR characterizations support the molecular structure of Oleuropein. Electrochemical and gravimetric tests were used to evaluate the corrosion inhibition capabilities of Oleuropein. According to polarization investigation, Oleuropein is a mixed-type inhibitor. Oleuropein's inhibitory efficacy increases with concentration, attaining an optimum value (98.92%) at 100 mg L^−1^. At high temperatures, Oleuropein can be considered an efficient inhibitor. Thermodynamic variables for the activation operation and copper dissolution were computed and addressed as well. Scanning electron microscopy (SEM) and energy dispersive X-ray (EDX) examinations revealed that Oleuropein produced an outer layer on the copper surface, shielding it from severe acid damage. Quantum chemical simulations were employed to propose molecular explanations for Oleuropein's inhibitory actions.

## Introduction

Heat exchangers made of copper metal and alloys are essential components for the thermal desalination sector, whether for desalting or heat restoration objectives to boost thermal performance^1^. The majority of industrial heat exchangers directly interact with a saltwater cooling solution, which causes scaling^2^. The need to disconnect machines for acid cleaning, which is usually dependent on acids, mandates a suitable method with careful control^3,4^. Acid cleaning procedures are utilized for de-scaling and the accompanying base metal preservation. Sulfuric acid treatment is commonly employed in the metals cleaning procedure to remove dust and scale from the base metal^5,6^.

The many corrosion problems that might occur during acid cleaning can lead to the breakdown of copper alloy heat exchanger^7,8^. Adding corrosion inhibitors into acid solutions is a cost-effective and efficient strategy to keep metals from corroding^9–12^. Organic corrosion inhibitors are widely utilized because of their inexpensive cost and high corrosion resistance. Luo et al.^13^ produced and developed a novel type of pyridazine-based compound as a copper corrosion inhibitor in 0.5 M sulphuric acid. At 298 K, this inhibitor has a maximal inhibitory effectiveness of 94.1%. Laggoun et al.^14^ investigated the inhibitory influences of *p*-toluenesulfonylhydrazide on copper corrosion in acid solution, demonstrating that it has a maximum anti-corrosion performance of greater than 90%. Many widely used organic corrosion inhibitors, on the other hand, have complicated synthesis procedures, severe toxicity, and are susceptible to pollution problems.

The approach of investigating aqueous extracts as corrosion inhibitors has a significant benefit. The key properties that enable the extracts to be the most successful recent the corrosion inhibitors categories are its nontoxicity, ecologically benign attitude, as well as the feature that they are affordable and sustainable. Previous research by Oukhrib et al.^15^ employed natural plant extracts including saffron extract as a copper corrosion inhibitor in the saltwater surrounding, with an inhibitory efficacy of 84% using 2 g/L. Jmia et al.^16^ investigated the inhibition activity of jujube pulp extract on copper corrode in 1 M HCl solution. The findings confirmed that the inhibition gradually increases with the amount of jujube pulp extract, achieving a highest of 93% at a dosage of 1 g/L.

In general, most extracts employed as corrosion inhibitors include a high concentration of organic components. The inhibitory activities of these extracts are caused by the total of the extract's constituents. The isolation of Oleuropein from olive leaf extract and analysis of its anticorrosion activity for copper in 1.0 M H_2_SO_4_ solution is a new trend in this work. In addition to theoretical research, we utilized chemical, electrochemical, and surface experiments to investigate Oleuropein's anticorrosion capabilities.

## Experimental part

### Materials

Copper samples with a purity of 98% were used in this investigation. Prior to testing, the copper sample was sanded by succession of emery papers (ranging from 600.0 to 1200.0 grades) and then cleaned with purified water and ethyl alcohol.

For all investigations, 1.0 M H_2_SO_4_ solutions were prepared using analar grade H_2_SO_4_ (Merck) and deionized water.

### Extraction, purification and characterization of Oleuropein from *Olea europaea* leaves

In the current work, the corrosion inhibition is caused by pure Oleuropein. The process for isolating and purifying Oleuropein from the extract's components was demonstrated in the next section.

50 g of freshly picked olive leaves (*Olea europaea* L.) were cut into small pieces that were gently boiled for 2 h in a 1 L beaker containing distilled water by using a mass ratio 1:10 (leaves/water). 250 mL of decoction, cooled at room temperature, were extracted with 50 mL of chloroform for three times, adding NaCl salt to facilitate the breaking of the emulsion. Pure Oleuropein was isolated extracting the aqueous phase with three aliquots of 50 mL of ethyl ethanoate. The organic fractions were collected, dehydrated with anhydrous sodium sulphate and then filtered. After the solvent evaporation under vacuum, 1.09 g of a sticky solid was obtained. A sample of the obtained sticky solid residue was dissolved in acetonitrile, then filtered and examined by High-Performance Liquid Chromatography (Agilent 1100 series, USA).

The qualitative evaluations were performed using (6540) quadrupole-time-of-flight (QTOF) mass analyzer equipped with electro-spray-ionization (ESI) device. FTIR analysis was taken using Jasco/FTIR/430 spectrometer fitted with an ATR crystal sampler.

^1^H-NMR and ^13^C-NMR spectra were acquired at ambient temperature in CD3OD using NMR spectrometer (Bruker Avance 400), and chemical shifts have been presented respect to TMS.

We confirm that all methods were performed in accordance with the relevant guidelines and regulations.

### Electrochemical experiments

A potentiostat/galvanostat/Gamry-model 3000 was used for the electrochemical experiments. The experiments were implemented out in a multiple cell, with a copper disc serving as the working part (having an effective surface area of 0.545 cm^2^), a Pt strip serving as the counter part, and a saturated calomel electrode (SCE) as the reference part. Varying potential ranges (± 250 mV/SCE vs. OCP) were applied to the copper electrode using 1.0 mV s^−1^ scan rate to produce the Tafel polarization plots. The results for electrochemical impedance spectroscopy (EIS) were taken at OCP at amplitude of 20 mV within a frequency region 100 kHz–0.1 Hz. To confirm the correctness of the electrochemical experiments, they were performed three times.

### Gravimetric experiments

To evaluate gravimetric measures, copper plates were sliced into 2.5 cm × 1.2 cm × 0.05 cm pieces (the average weight = 1.4534 g). The copper plates were submerged in 100 mL of either 1.0 M H_2_SO_4_ or Oleuropein-treated H_2_SO_4_ solutions. The gravimetric was calculated using the standard method G1-03-2017-e1 ASTM^17^. For 24 h, the samples were submerged in solutions. The trials were carried out in three replicate, and the mean mass loss was determined. Numerous experiments at various temperatures (298, 313, 323, and 333 K) utilizing a temperature-controlled water bath were planned.

### Surface morphology analysis

SEM (JEOL JEM-1200EX) combined with energy dispersive X-ray spectrometry was employed to inspect the surface aspect of a copper plate submerged in experiment liquids for 24 h.

### Theoretical considerations

To study the interaction activities of Oleuropein, quantum chemical simulations based on the DFT approach were done. Geometry optimization was performed utilizing VAMP configuration of Accelrys Inc.'s Materials Studio-6.0.

## Results and discussion

### Oleuropein characterizations

**Figure 1:**
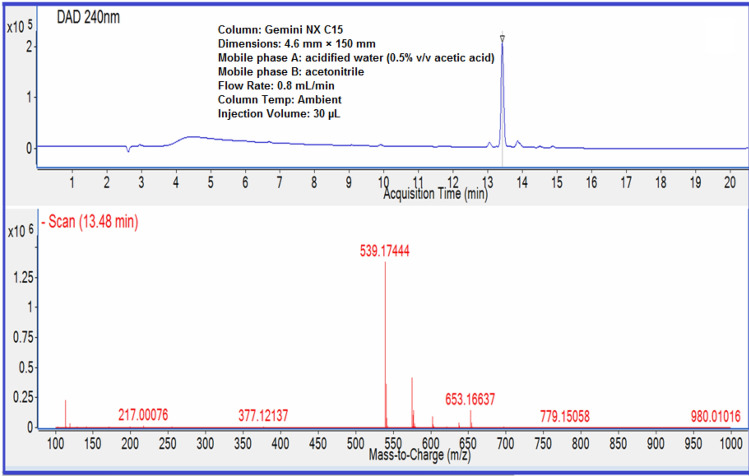
HPLC–DAD chromatogram and mass spectrum of Oleuropein.

The Oleuropein used in this work has been characterized giving analytical and spectral data. The HPLC–DAD chromatogram of purified sample and its ESI–MS profile are shown in Fig. 1. The existence of a molecular ion (m/z 539) and a fragment deriving from the decline of the sugar molecule, matching to the aglycon (m/z 377) is confirmed by the mass spectrum.

**Figure 2:**
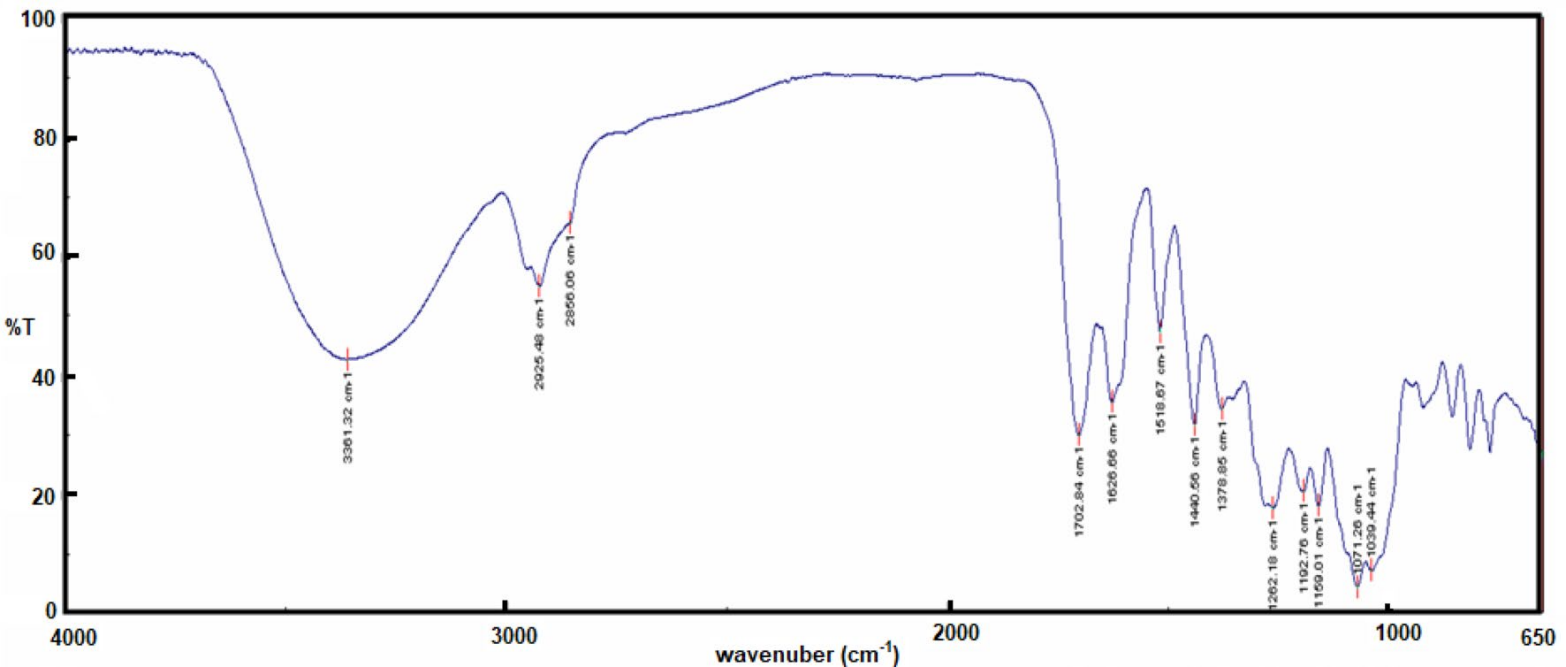
FT-IR spectrum of Oleuropein.

The FT-IR spectrum performed for Oleuropein extract was seen in Fig. 2. The OH stretching vibrations cause a wide band (3700–3100 cm^−1^). C–H stretching bands appear at 2926 and 2856 cm^−1^. The 1750–1500 cm^−1^ area correlates directly to stretching vibrations of C=O and C=C. In the complex region of 1500–1200 cm^−1^ appears the C–O stretching that produces an absorption band 1262 cm^−1^.

^1^H NMR (400 MHz, CD_3_OD): δ = 7.51 (s, 1H, s), 6.85–6.67 (m, 2H), 6.54–6.50 (m, 1H), 6.08 (1H, m), 5.91 (br. S, 1H), 4.22 (m, 2H, m), 4.00 (m, 2H, m), 3.73 (s, 3H, s), 3.67–3.30 (m), 2.80–2.40 (4H, m), 1.66 (3H, d, J = 7.1 Hz) ppm.

^13^C NMR (100 MHz, CD_3_OD): δ = 172.35, 167.75, 154.24, 145.20, 143.87, 130.80, 129.78, 123.97, 120.39, 116.12, 115.07, 108.34, 99.88, 94.23, 77.35, 76.88, 73.73, 70.44, 66.00, 61.70, 51.06, 40.29, 34.37, 30.81, 12.63 ppm.

All the experimental data related with the characterization LC–MS, FT-IR, ^1^H and ^13^C-NMR related with the Oleuropein used in this work are in agreement with the literature^18,19^.

### Polarization and EIS measurements

**Figure 3:**
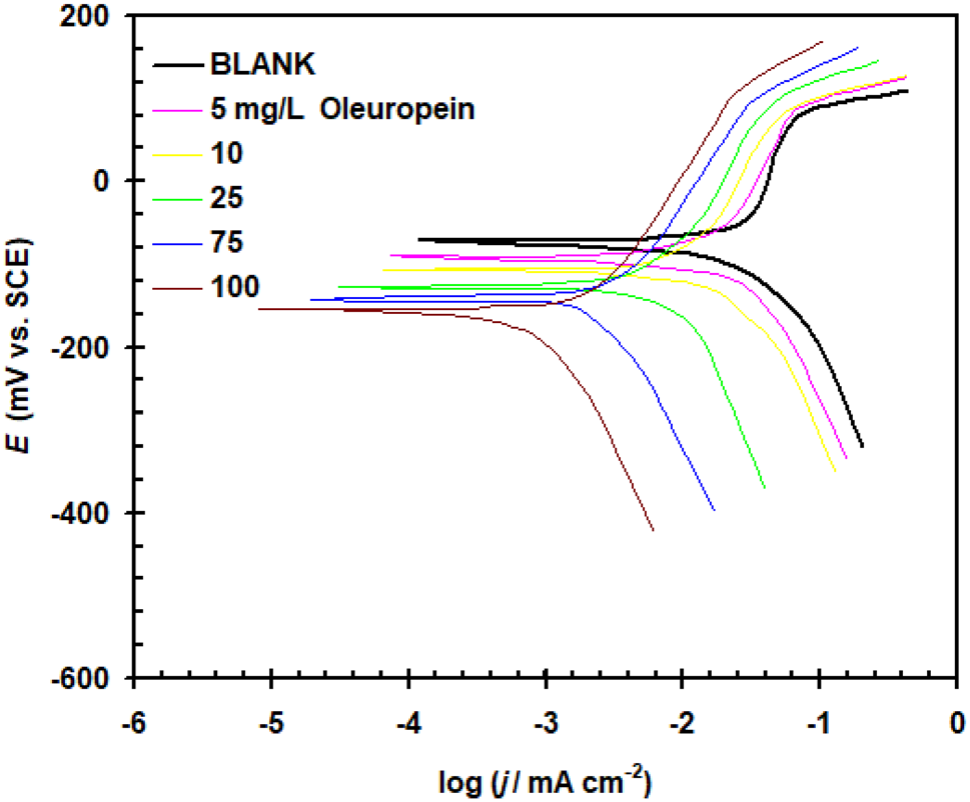
Tafel polarization plot for copper in 1.0 M H_2_SO_4_ in the absence and addition of varying concentration levels of Oleuropein at 298 K.

At 298 K, Tafel polarization plot for copper in 1.0 M H_2_SO_4_ in the presence of varying concentration levels of Oleuropein is provided in Fig. 3. The polarization evaluation showed that increasing the quantity of Oleuropein alters both the cathodic and anodic current.

Tafel plot revealed the presence of corrosion breakdown potential at almost + 75 mV due to passivity breakdown^20^. The observed critical breakdown potential of copper increases as the concentration of Oleuropein increases. The Tafel polarization data are provided in Table 1. The change in corrosion potential (*E*_corr_) readings shows no recognizable sequence. The gap in *E*_corr_ value for both blank acid solution and acid solution containing the Oleuropein seemed to be less than 85 mV, supporting the Oleuropein's mixed type behavior^21,22^. By increasing the concentration of Oleuropein, the *E*_corr_ value moved also towards the negative direction. This change is caused by a decrease in the hydrogen evolution process on the copper surface resulting from Oleuropein molecule adsorption^23^. There have been no notable changes in the cathodic Tafel slop (β_c_) and anodic Tafel slop (β_a_) were detected, showing that adding Oleuropein to the corrosion environment had no effect on the mechanism of the anodic and cathodic processes. In the presence of Oleuropein, the corrosion current density (*j*_corr_) values reveal a significant decrease (*j*_corr_ = 31.54 μA cm^−2^ for blank and 0.34 μA cm^−2^ for Oleuropein 100 mg L^−1^). This implies that Oleuropein suppresses copper electrode corrosion in 1.0 M H_2_SO_4_ solutions^24^. The inhibition ability of Oleuropein (*η*_j_%) was quantified utilizing the formula below^25,26^:

1 $$ \eta_{{\text{j}}} \% = \frac{{j_{{{\text{corr}}(0)}} - j_{{{\text{corr}}}} }}{{j_{{{\text{corr}}(0)}} }} \times 100 $$

where *j*_corr(0)_ denotes the corrosion current densities measured in an acid solution without Oleuropein.

**Table 1:** Polarization parameters for Cu in 1.0 M M H_2_SO_4_ solution in the absence and presence of Oleuropein at 298 K.

Oleuropein (mg L^−1^)	*E*_corr._ mV (SCE)	*j*_corr._ (μA cm^−2^)	β_a_ (mV dec^−1^)	− β_c_ (mV dec^−1^)	*θ*	*η*_j_%
Blank	− 72	31.54	121	265	–	–
5	− 91	25.11	117	254	0.2038	20.38
10	− 108	18.82	106	232	0.4032	40.32
25	− 128	7.67	123	244	0.7568	75.68
75	− 142	2.05	129	201	0.9350	93.50
100	− 155	0.34	104	286	0.9892	98.92

Oleuropein's inhibition ability goes up with concentration, achieving a maximum output (98.92%) at 100 mg L^−1^ with surface coverage ($\theta = 1 - \frac{{j_{{{\text{corr}}}} }}{{j_{{{\text{corr}}(0)}} }}$) reaches to 0.9892. These data corroborate that Oleuropein has a strong inhibitory effect on copper corrosion in 1.0 M H_2_SO_4_.

**Figure 4:**
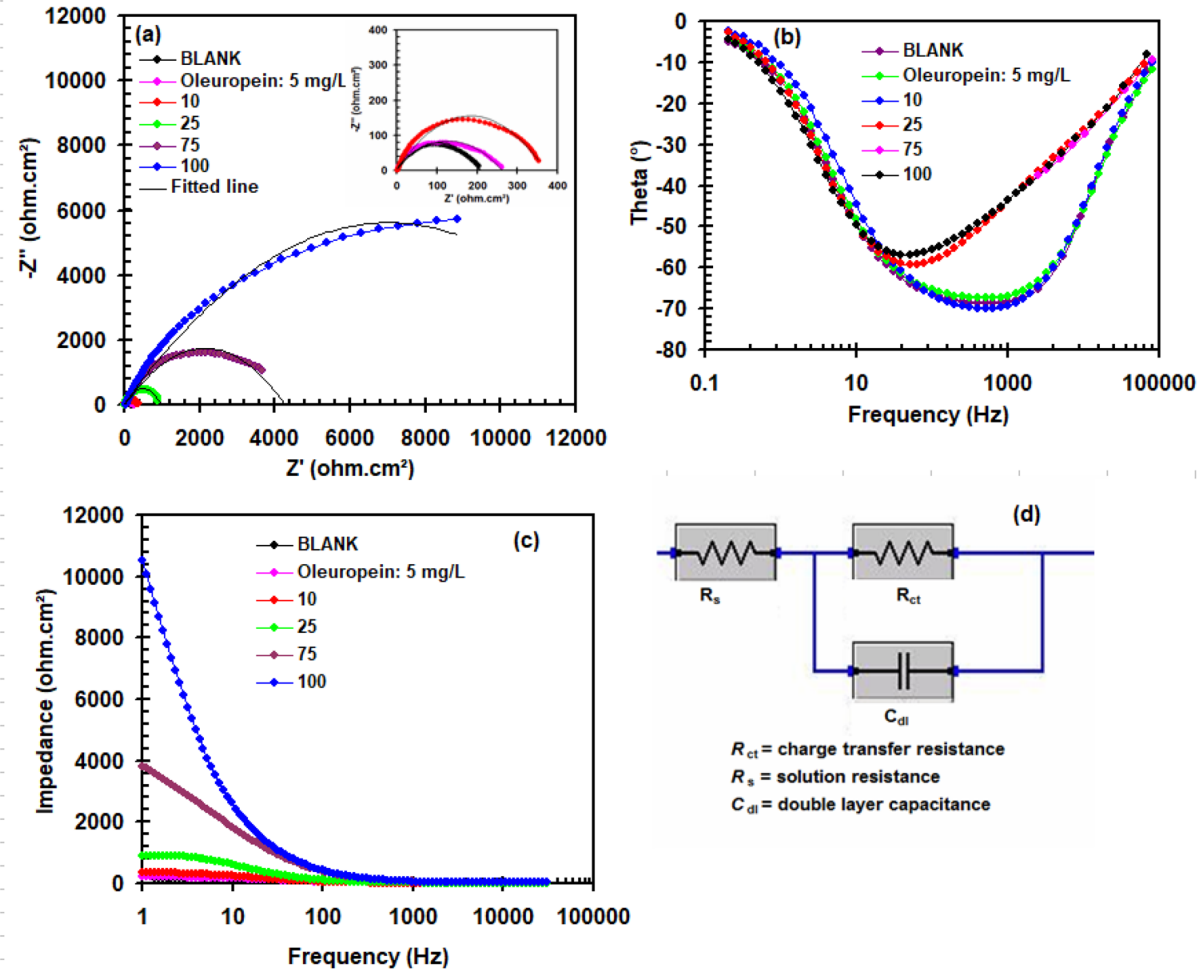
(**a**) Nyquist, (**b**) phase angle, (**c**) modulus plots and (**d**) the equivalent circuit for copper in 1.0 M H_2_SO_4_ in the absence and addition of varying concentration levels of Oleuropein at 298 K.

The resultant Nyquist, phase angle and modulus plots can be illustrated in Fig. 4a–c, respectively. The equivalent circuit designed to estimate impedance behavior can also be seen in Fig. 4d. The generated impedance plots (Fig. 4a) exhibit flattened shapes (loop capacitive) that correlate to charge transfer^27,28^. The uniformity and surface topography of the copper surface cause this loop capacitive depression^29^. The radius of the capacitive loop grew in ways consistent with the amount of Oleuropein. The creation of an Oleuropein layer prevents the production of the corrosion product^30,31^. The addition of Oleuropein causes the phase angle values (Fig. 4b) to increase, resulting from the creation of a thicker protective layer. The EIS variables derived after fitting the curves in the equivalent circuit are summarized in Table 2. The charge transfer resistance (R_ct_) increases while the double layer capacitance (C_dl_) magnitude drops when the concentration of Oleuropein increases. The increase in R_ct_ value, which reaches a value of 12,000 cm^2^ at a concentration of 100 mg L^−1^, is predominantly due to Oleuropein adsorption on the copper surface^32–34^. When the concentration of Oleuropein inside the acid solution was increased, the value of the C_dl_ decreases to 1.33 × 10^–6^ F cm^−2^ as particularly in comparison to the blank value of 5.99 × 10^–5^ F cm^−2^ attributable to the restricted accessibility of charged particles towards the surface^35^. The inhibition ability of Oleuropein (*η*_R_%) was quantified utilizing the formula below^36^:

2 $$ \eta_{{\text{R}}} \% = \frac{{R_{{{\text{ct}}}} - R_{{{\text{cto}}}} }}{{R_{{{\text{ct}}}} }} \times 100 $$

where *R*_cto_ denotes the charge transfer resistance measured without Oleuropein. The inhibitory effectiveness rises with the concentration of Oleuropein, reaching a maximum (98.35%) at 100 mg L^−1^. This means that Oleuropein molecules are adsorbed at the copper/solution interface, delaying the oxidation reaction.

**Table 2:** EIS parameters and corresponding inhibition efficiency for for Cu in 1.0 M M H_2_SO_4_ solution in the absence and presence of Oleuropein at 298 K.

Oleuropein (mg L^−1^)	Rct (Ω cm^2^)	C_dl_ (F cm^−2^)	*η*_R_%
Blank	198	8.04E−05	
5	265.77	5.99E−05	25.5
10	339.62	4.69E−05	41.7
25	889.89	1.79E−05	77.75
75	3413.79	4.66E−06	94.2
100	12,000	1.33E−06	98.35

### Gravimetric measurements

The mass loss approach is a non-electrochemical that represents dipping copper pieces in 1.0 M H_2_SO_4_ for an extended duration of time. The corrosion rate (*C*_R_) was evaluated using the next expression^37^:

3 $$ C_{R} = \frac{W}{A \times t} $$

*A* = copper piece surface area (cm^2^), *t* = duration (h), *W* = mass loss (mg).

The inhibition ability of Oleuropein (*η*_W_%) was quantified utilizing the formula below^37^:

4 $$ \eta_{{\text{W}}} \% = \frac{{C_{{{\text{R}}0}} - C_{{\text{R}}} }}{{C_{{{\text{R}}0}} }} \times 100 $$

where *C*_R0_ denotes the corrosion rate measured in an acid solution without Oleuropein. Table 3 summarizes the *C*_R_ and *η*_W_% at various Oleuropein concentrations. The value of *C*_R_ continues to decrease as the amount of Oleuropein increases. This reduction in *C*_R_ with increasing Oleuropein concentration reflects an increasing trend in surface covering on the copper via Oleuropein molecules. For a concentration of 100 mg L^−1^ of Oleuropein, the highest inhibitory effectiveness (*η*_W_% = 96.20) was found.

**Table 3:** Gravimetric analysis for Cu in 1.0 M M H_2_SO_4_ solution in the absence and presence of Oleuropein at 298 K.

Oleuropein (mg L^−1^)	C_R_ (μg cm^−2^ h^−1^)	*η*_*W*_%
Blank	43.65 ± 1.32	
5	31.25 ± 1.25	28.4
10	23.47 ± 0.98	46.23
25	11.84 ± 0. 54	72.87
75	3.59 ± 0.32	91.78
100	1.66 ± 0.26	96.2

### Temperature effects and thermodynamic analysis

Temperature is an important factor in the research of the corrosion mechanism in broad sense. Also, because rising temperatures have an impact on the interaction between the solution and the metal^38,39^. This allows us to evaluate the manner of the Oleuropein's adsorption on the copper surface and determine the Oleuropein's stability as the temperature rises**.** Table 4 illustrates the corrosion rate and inhibition capacity for Cu in 1.0 M H_2_SO_4_ solution with and without Oleuropein (100 mg L^−1^) as a function of temperature (298–333 K). The data show that the *C*_R_ of copper in acid solution (either controlled or inhibited) tends to increase as temperature rises. This trend may be understood by the rough of the surface of the copper caused by the elevated temperature, as well as a switch in the adsorption/desorption balance toward desorption of Oleuropein from the copper surface^40^. The *η*_*W*_% steadily drops as temperature rises (Table 4), indicating a physisorption process^41^. Because rising temperatures have little effect on *η*_*W*_%, this signifies that the Oleuropein/surface system is stable at high temperature levels. Particularly at high temperature, Oleuropein can be regarded as an effective inhibitor.

**Table 4:** Gravimetric data at different temperatures for Cu in 1.0 M H_2_SO_4_ solution in the presence/absence of Oleuropein (100 mg L^−1^).

Temperature (K)	Oleuropein	*C*_*R*_ (μg cm^−2^ h^−1^)	*η*_*W*_ (%)
298	0	43.65 ± 1.32	–
	+	1.66 ± 0.26	96.20
313	0	49.76 ± 1.70	–
	+	3.05 ± 0.29	93.87
323	0	55.16 ± 2.06	–
	+	5.38 ± 0.33	90.24
333	0	70.35 ± 2.69	–
	+	8.49 ± 0.32	87.93

**Figure 5:**
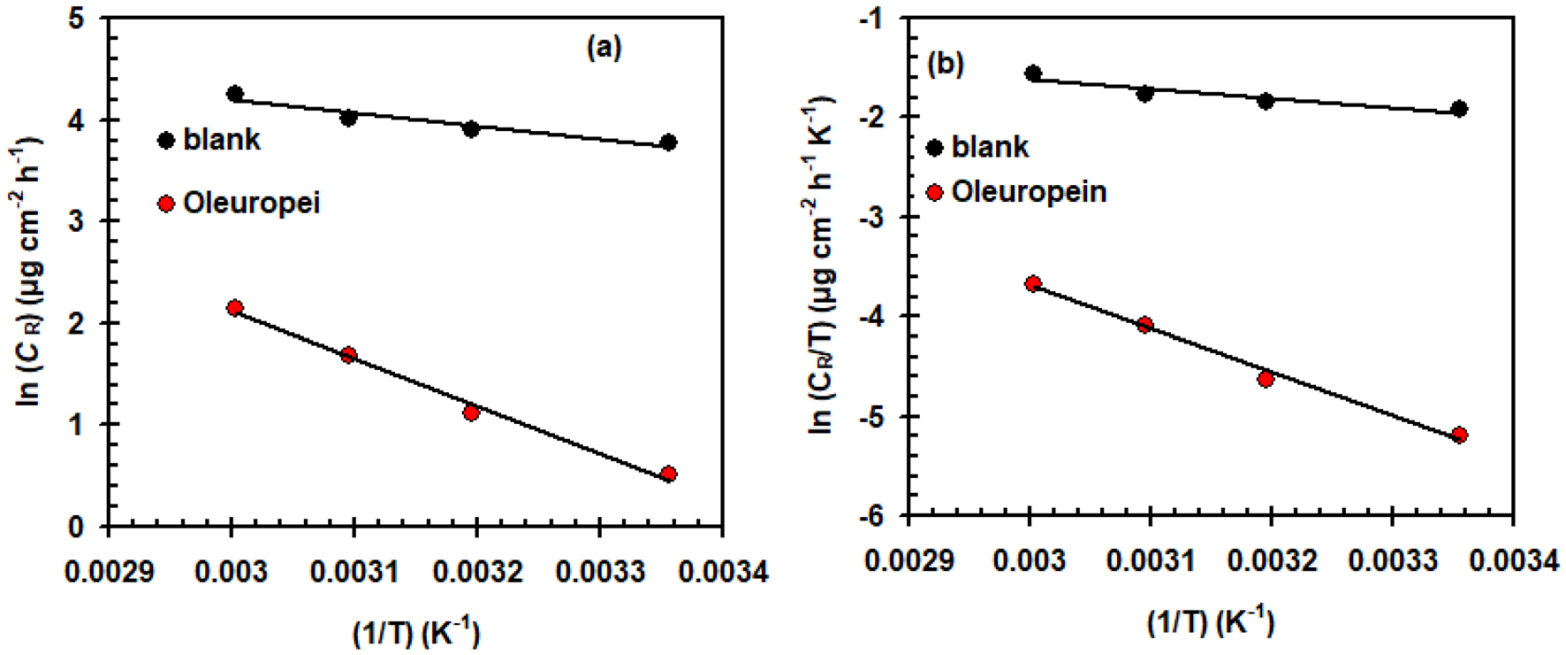
Arrhenius (**a**) and transition state (**b**) plots for copper in 1.0 M H_2_SO_4_ solution in the presence/absence of Oleuropein (100 mg L^−1^).

The examination of the *C*_R_ charting as a temperature—dependent allowed for the estimation of different variables including the activation energy (*E*_a_), enthalpy change (ΔH^*^), and entropy change (ΔS^*^) to describe the oxidation operation and the likely mechanism of inhibitor adsorption. The Arrhenius plot (Fig. 5a) was used for evaluating the *E*_a_ in the presence/absence of Oleuropein (100 mg L^−1^), utilizing the below formula^42^:

5 $$ C_{R} = Ae^{{\frac{{ - E_{a} }}{RT}}} $$

*R* = molar gas constant, *T* = Kelvin temperature and *A* = pre-exponential constant.

The inclusion of Oleuropein raises the *E*_a_ from 10.64 kJ mol^−1^ (blank solution) to 38.79 kJ mol^−1^ (100 mg L^−1^ Oleuropein). Copper corrosion is slowed by high activation energy in the presence of Oleuropein. The adsorption of Oleuropein on the surface of copper causes a rise in the width of the double layer, which raises the energy barrier necessary to initiate the corrosion reaction. This was linked to the favorable physical sorption of Oleuropein molecules^12^.

The transition state equation and plot (Fig. 5b) was used to derive the values of ΔH^*^ and ΔS^*^:

6 $$ C_{R} = \frac{RT}{{Nh}}\exp \left( {\frac{{\Delta S^{ * } }}{R}} \right)\exp \left( {\frac{{ - \Delta H^{*} }}{RT}} \right) $$

*N* = 6.2022 × 10^23^ mol^−1^ and *h* = 6.6261 × 10^−34^ m^2^ kg s^−1^.

The inclusion of Oleuropein raises the ΔH* from 8.02 kJ mol^−1^ (blank solution) to 36.18 kJ mol^−1^ (100 mg L^−1^ Oleuropein). The endothermic character of copper oxidation in acid solution can be seen from the positive magnitude of ΔH*^43^. The ΔS* varied to a slight extent from − 186.86 (blank solution) to − 119.66 J mol^−1^ K^−1^ (100 mg L^−1^ Oleuropein). Besides that, the moving from a negative value in case of blank solution to a less negative value of ΔS* in the case of the solution containing 100 mg L^−1^ Oleuropein could be likened to the freedom of a considerable portion of some more dis-ordered H_2_O molecules which have been adsorbed on the copper surface and are being replaced by more ordered Oleuropein molecules^44^.

The Langmuir isotherm model (Eq. 7) is mostly used to verify adsorption for this system^45^.

7 $$ \frac{{C_{{{\text{inh}}}} }}{\theta } = \frac{1}{{K_{{{\text{ads}}}} }} + C_{{{\text{inh}}}} $$

*C*_inh_ = Oleuropein concentration and *K*_ads_ = equilibrium constant.

**Figure 6:**
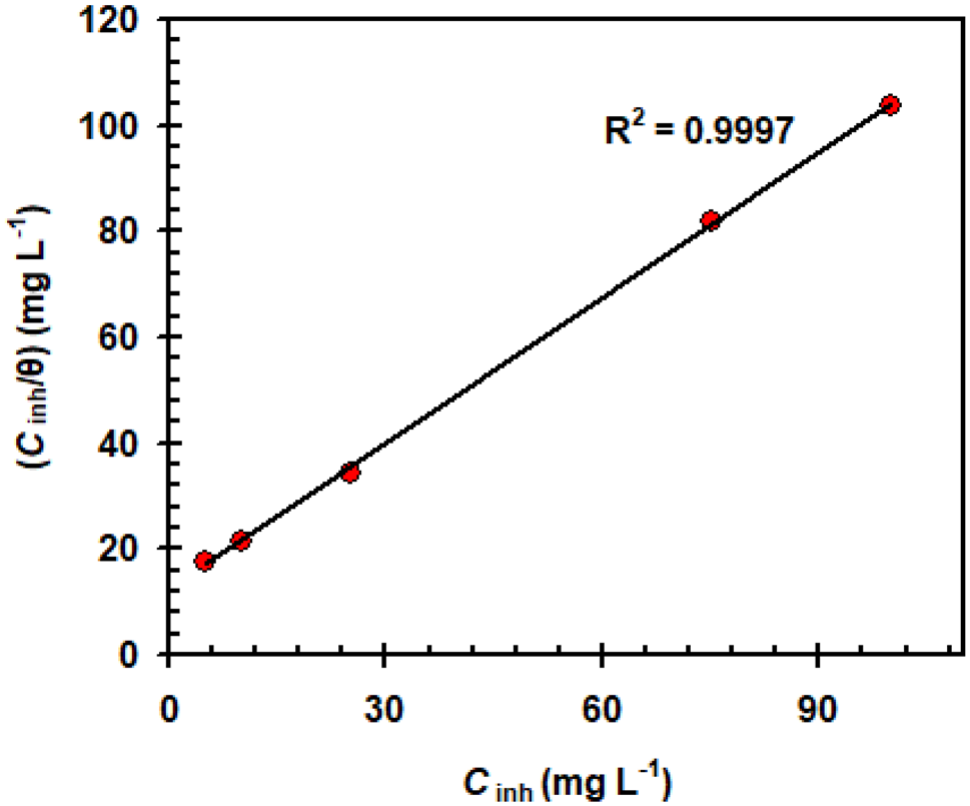
Langmuir adsorption isotherm of Oleuropein.

Figure 6 illustrates the Langmuir isotherm for Oleuropein. The correlation coefficients (R^2^) in Fig. 6 are much closer to one (i.e. 0.9997), demonstrating that this approach is acceptable for determining adsorption ability^46^. Importantly, the minimum *K*_ads_ value (i.e. 0.0806 L mg^-1^) reflects Oleuropein's physical adsorption features. The Gibbs free energy (∆G_ads_°) of an adsorption system is calculated as follows^47^:

8 $$ \Delta {\text{G}}_{{{\text{ads}}}}^\circ = \, - RTLn\left( {55.5K_{{{\text{ads}}}} } \right) $$

∆G_ads_° has a value of − 36.3 kJ mol^−1^, indicating that Oleuropein adsorption is mostly a physisorption mechanism^47^.

### SEM/EDX analysis

**Figure 7:**
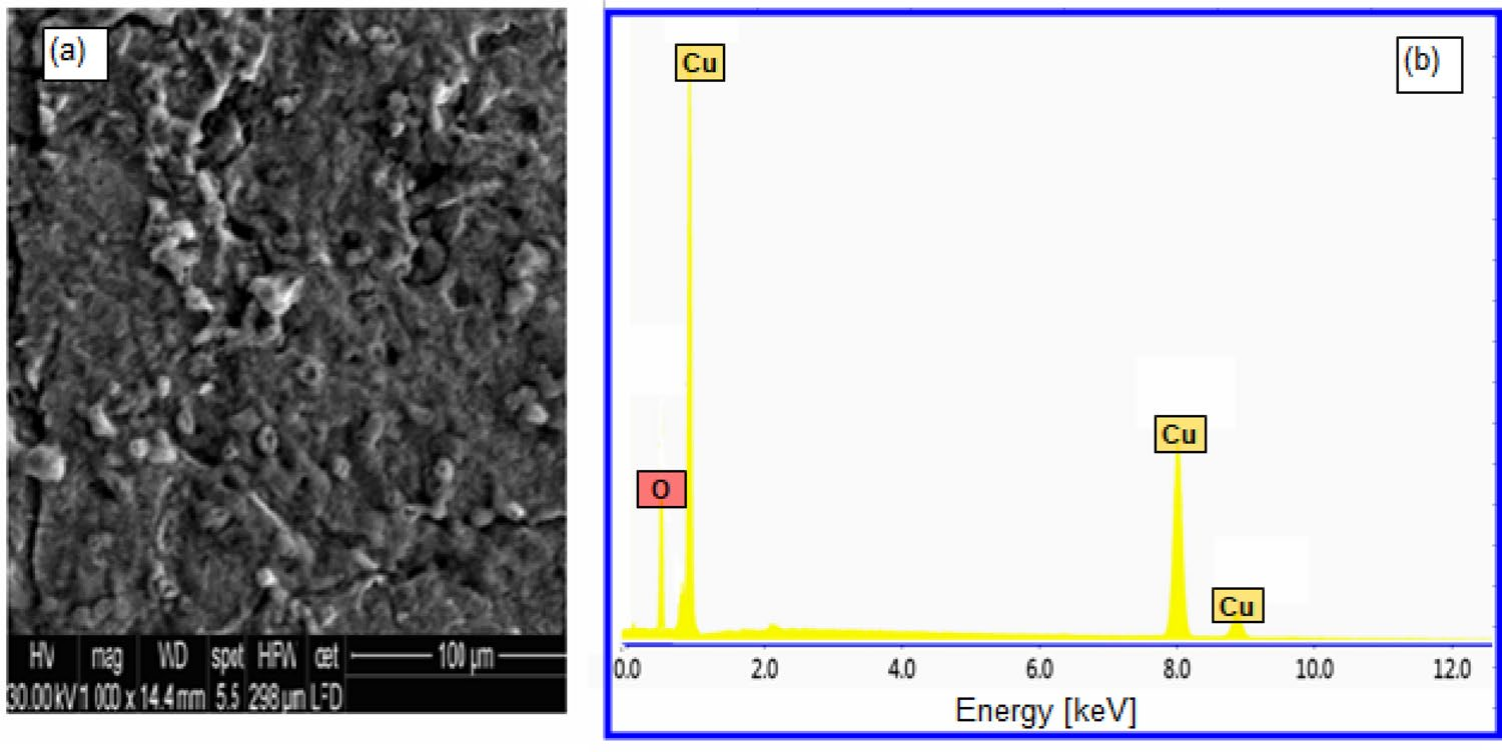
(**a**) SEM and (**b**) EDX for copper in 1.0 M H_2_SO_4_ at 298 K.

**Figure 8:**
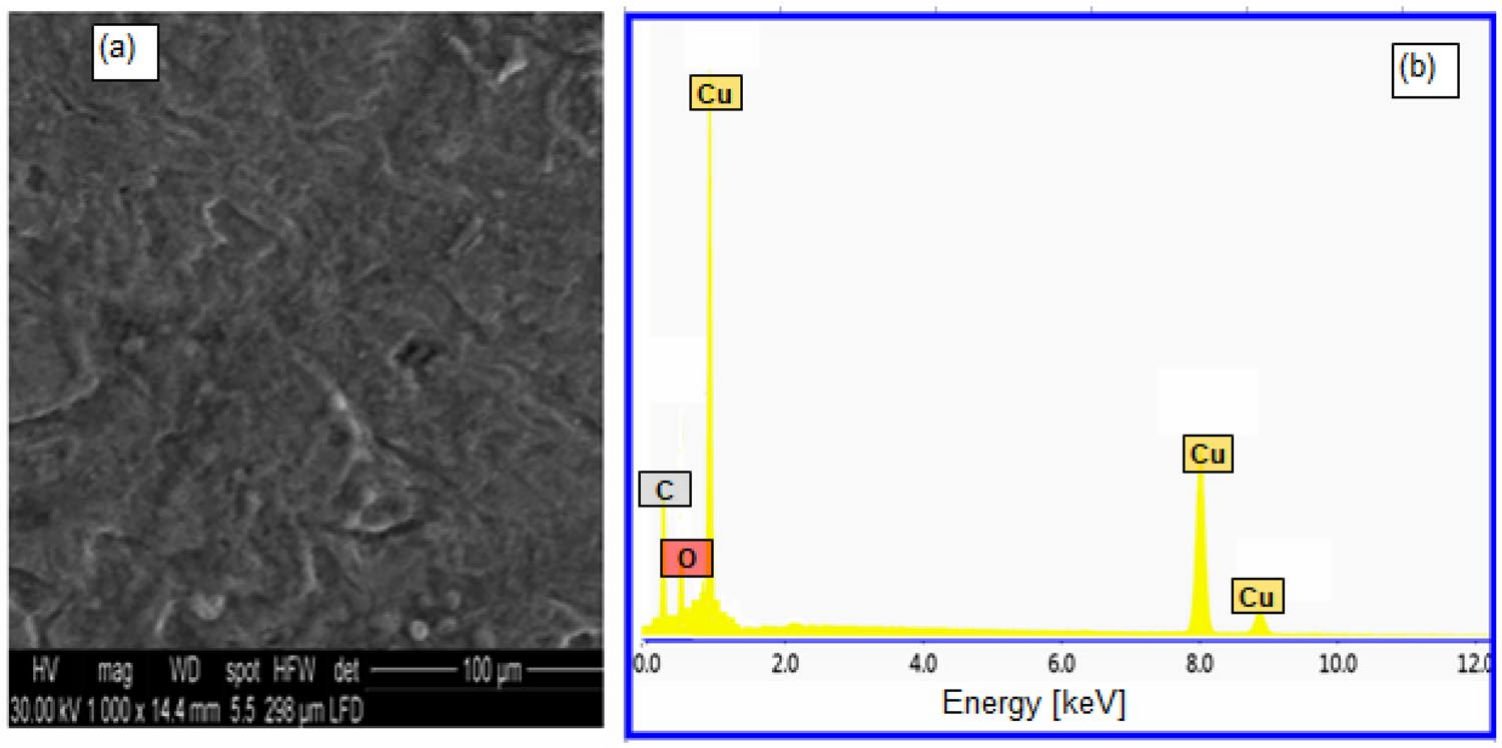
(**a**) SEM and (**b**) EDX for copper in 1.0 M H_2_SO_4_ containing Oleuropein (100 mg L^−1^) at 298 K.

To support the data of the electrochemical and gravimetric tests, observational microscopic SEM investigations were combined with quantification EDX analysis. A SEM top photo of the surface of copper in 1.0 M H_2_SO_4_ solution without Oleuropein is shown in Fig. 7a, and it can be seen that the uneven corrosion of the entire copper surface is very dense and clear. The EDX examination for the blank solution (Fig. 7b) revealed the existence of Cu and O signals, which are corrosive elements for copper oxide and so support the corrosion of copper. The addition of 100 mg L^−1^ of Oleuropein to 1.0 M H_2_SO_4_ solution reduces the corrosion extent on the copper surface and forms a nonuniform covering layer on the copper surface, as seen in the SEM picture (Fig. 8a). The appearance of C and O signals in the EDX spectra, which represent the primary constituents of Oleuropein molecule, is also shown in Fig. 8b. These findings demonstrate the creation of a protective layer when the tested Oleuropein interacts with the copper surface in acidic media.

### Mechanism and theoretical considerations

The dissolving of copper in acidic media was carried out in the following stages^48,49^:

9 $$ {\text{Cu }} = {\text{ Cu}}^{ + }_{{({\text{adsorbed species}})}} + {\text{ e }}\left( {{\text{fast step }} + {\text{ at the anode site}}} \right) $$

10 $$ {\text{Cu}}^{ + }_{{({\text{adsorbed species}})}} = {\text{ Cu}}^{{{2} + }}_{{({\text{soluble species}})}} + {\text{ e }}\left( {{\text{slow step }} + {\text{ at the anode site}}} \right) $$

11 $$ {\text{O}}_{{2}} + {\text{ 4H}}^{ + } + {\text{ 4e }} = {\text{ 2H}}_{{2}} {\text{O }}\left( {\text{at the cathode site}} \right) $$

**Figure 9:**
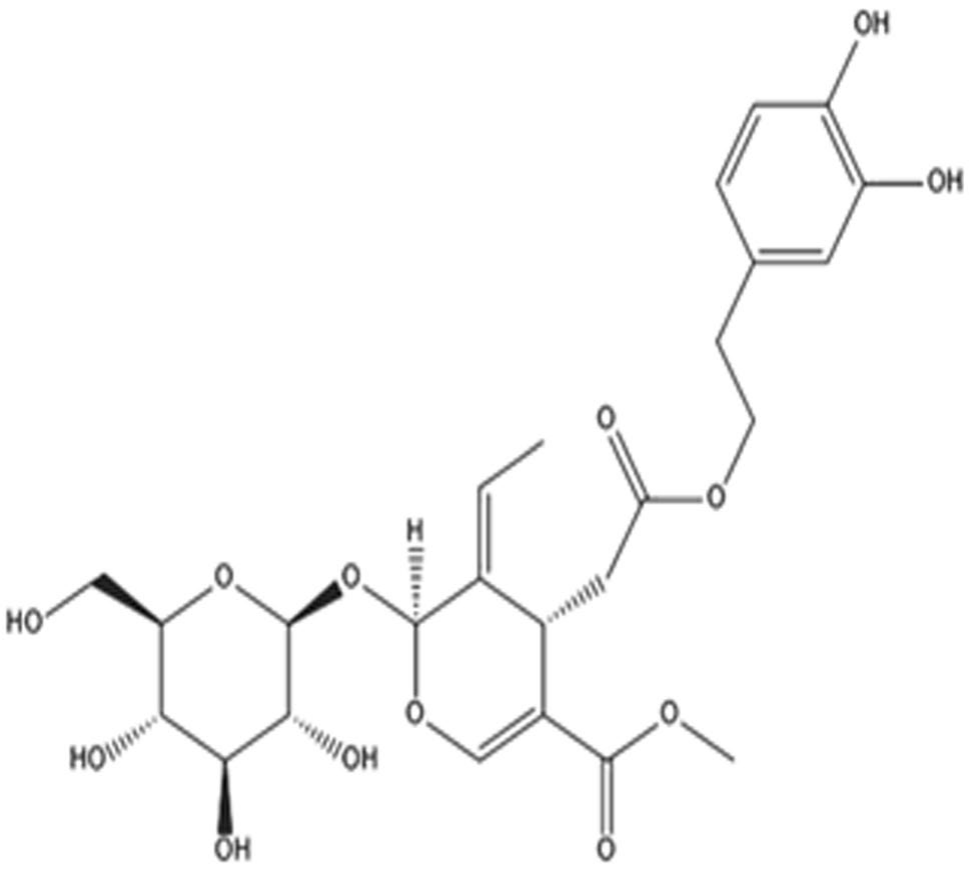
Chemical structure of Oleuropein.

**Figure 10:**
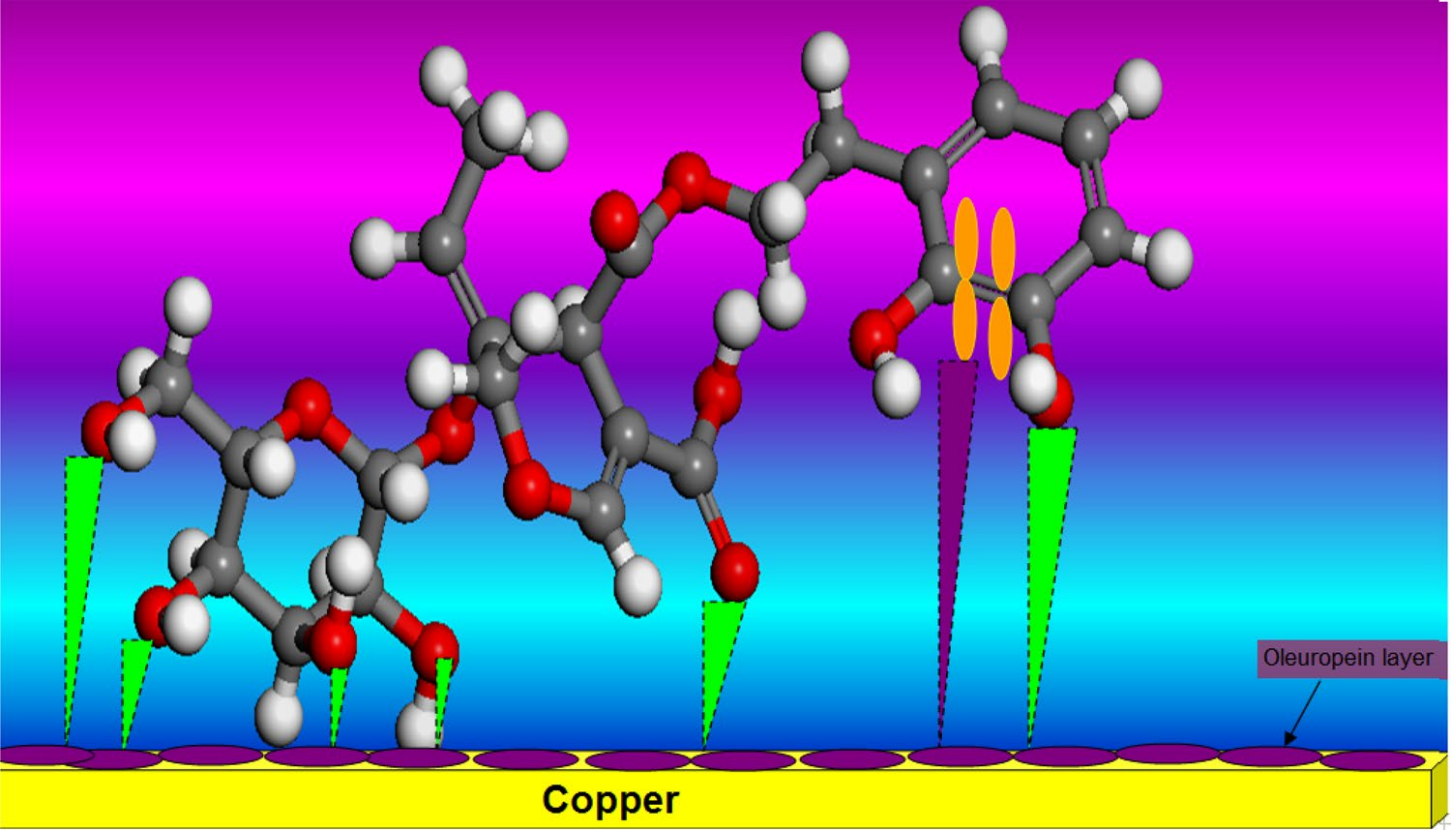
Schematic illustration of inhibition mechanism of Oleuropein adsorption on copper in 1.0 M H_2_SO_4_ yielding the formation of protective layer.

The barrier protection copper oxide layers may be quickly solubilized at low pH values, based on the potential–pH chart^50^. The various findings (SEM/EDX) demonstrate that the adsorption of the Oleuropein molecule on the copper surface is the primary mechanism of corrosion prevention. At the surface of copper, the Oleuropein molecule comprises O-bearing hydroxyl groups (see Fig. 9) which possess a great affinity for Cu. In parallel to the attachment of aromatic rings, physisorption on the copper surface results through partial transfers of O electrons and the creation of double bonds (see Fig. 10). The Oleuropein compound adsorbed film functions as a shield between both copper surface and the acidic corrosive liquid^51–53^. Another mechanism that may be included is the creation of Cu(I)-Oleuropein complexes on the copper surface. This complex suppresses the anodic process^54^.

**Figure 11:**
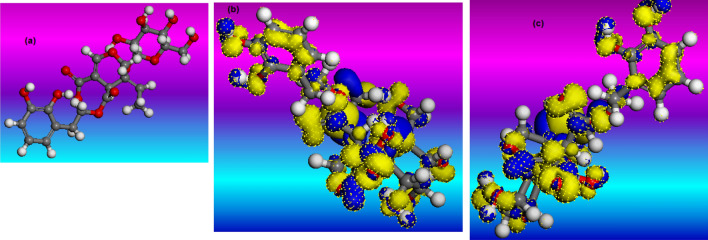
(**a**) optimized geometrical arrangement, (**b**) HOMO and (**c**) LUMO of Oleuropein.

To determine the molecular activity of Oleuropein, quantum chemical simulations based on the DFT model were carried out. As a consequence, the chemical characteristics of Oleuropein can be seen in Fig. 11. Figure 11a depicts the whole optimized geometrical arrangement of Oleuropein. The HOMO orbital reflects the molecule's electron-donating ability (Fig. 11b), whereas the LUMO orbital reflects the molecule's electron-acquiring ability (Fig. 11c)^55^. The HOMO and LUMO electron clouds for Oleuropein are generally observed to be virtually entirely centred in aromatic ring groups and OH groups. This reveals that all these active adsorption groups may exchange electrons with copper to create covalent connections. Oleuropein has a dipole moment (*μ*) of 13.67 debye. Adsorption on the metal surface increases as a result of the high dipole moment^56^. The high HOMO energy value (*E*_HOMO_ = − 6.654 eV) draws attention to the capacity of the Oleuropein molecule to associate with the copper surface. Furthermore, the low LUMO energy (*E*_LUMO_ = − 3.577 eV) alludes to the capacity of the Oleuropein molecule to receive electrons from the copper surface. Additionally, the low energy difference (ΔE = *E*_LUMO_ − *E*_HOMO_, 3.077 eV) correlates to Oleuropein's good inhibitory performance^57^.

Oleuropein's electronegativity (χ) and global hardness (η) properties are computed using the following relationships^58^:

12 $$ \chi \, = \, 0.{5 } \times \, \left( {{\text{I }} + {\text{ A}}} \right), \, \eta \, = \, 0.{5 } \times \, \left( {{\text{I }} - {\text{ A}}} \right) $$

I = ionization potential =  − *E*_HOMO_, A electron affinity =  − *E*_LUMO_.

5.115 eV and 1.538 eV are the estimated values for χ and η, respectively.

Oleuropein molecules with a high χ value have a strong capacity to capture electrons and, as a consequence, have a high adsorption capacity. Besides that, the small η value for Oleuropein molecules shows that the surface copper and the inhibitor Oleuropein have a strong interaction^59^.

The number of electrons transmitted (ΔN) was also computed using the following formula:

13 $$ \Delta N = \, \left[ {\left( {\chi_{{{\text{cu}}}} - \, \chi_{{{\text{inh}}}} } \right)} \right]/ \, \left[ {{2 }\left( {\eta_{{{\text{cu}}}} + \, \eta_{{{\text{inh}}}} } \right)} \right] $$

χ_cu_ = 4.48 eV, η_cu_ = 0 eV for copper^60^.

The inhibitory effectiveness improved with increasing electron-donating capability at the cooper surface because of ΔN < 3.6 (i.e. ΔN = − 0.206)^61^. Oleuropein was the electron donor in this experiment, while the copper surface was the acceptor.

## Conclusions

Oleuropein was isolated from olive leaf and examined for its anticorrosion capability for copper in 1.0 M H_2_SO_4_ solution utilizing gravimetric, electrochemical, SEM, and EDX studies. To confirm the experimental findings, quantum chemical simulations on the produced Oleuropein were also accomplished.

The investigation yielded the following conclusions:

- Many of the experimental data from LC–MS, FT-IR, ^1^H, and ^13^C-NMR analysis verify the molecular structure of Oleuropein.
- Oleuropein demonstrated significant corrosion protection for copper in 1.0 M H_2_SO_4_ solution.
- The efficiency of Oleuropein inhibition enhances with concentration and tends to decrease with temperature.
- Oleuropein's inhibitory power increases with concentration, reached its maximum (98.92%) at 100 mg L^−1^, and it also functions as a mixed type inhibitor.
- *R*_ct_, in specifically, achieves a value of 12,000 Ω cm^2^ at a dosage of 100 mg L^−1^ as compared to that recorded in the absence of Oleuropein (198 Ω cm^2^).
- Adding Oleuropein increases the *E*_a_ from 10.64 kJ mol^−1^ (blank solution) to 38.79 kJ mol^−1^ (100 mg L^−1^ Oleuropein). Furthermore, we found that the positive value of ΔH* and ΔS* varied somewhat from − 186.86 J mol^−1^ K^−1^ (blank solution) to − 119.66 J mol^−1^ K^−1^ (100 mg L^−1^ Oleuropein).
- SEM/EDX clearly shows the creation of an outer covering when the tested Oleuropein makes contact with the copper surface in acidic environments.
- Quantum chemical factors clearly demonstrate that Oleuropein has considerable corrosion inhibition strength, which is consistent with experimental data.
